# Bilateral camptodactyly

**DOI:** 10.11604/pamj.2022.42.239.36313

**Published:** 2022-07-28

**Authors:** Aditya Laxmikant Kekatpure, Aashay Kekatpure

**Affiliations:** 1 Orthopaedic Surgery, Jawaharlal Nehru Medical College, Datta Meghe Institute of Medical Sciences (DMIMS), Wardha, India,; 2 Orthopaedic Surgery, NKP Salve Institute of Medical Sciences and Research Centre and Lata Mangeshkar Hospital, Nagpur, India

**Keywords:** Camptodactyly, flexion contracture, clinodactyly

## Image in medicine

Greek for “camptodactyly” is “bent finger,” and Smith and Kaplan's exhaustive review lists all of its numerous synonyms (1968). It refers to a non-traumatic flexion contracture of the proximal interphalangeal joint, typically of the little finger, in surgical practise. A post-traumatic aetiology rather than camptodactyly is suggested by involvement of the distal interphalangeal joint or the metacarpo-phalangeal joint. Prevalence less than 1%. It can be unilateral (33%) or bilateral (66%), if bilateral, can be symmetric or asymmetric. Camptodactyly should not be confused with clinodactyly or Kirner's deformity. A 12-year-old child presented to the outpatient department with painless contracture of bilateral small finger at the level of proximal interphalangeal joint (PIP) with minimal involvement of the ring and middle finger. The deformity was noticed during the recent growth spurt of the child. On examination there was flexion contracture present in bilateral small finger at the level of PIP joint. As the contracture was passively correctable with PIP flexion less than 30 degrees, the patient was educated regarding the passive stretching and splinting. He is currently under regular follow up. If we notice worsening of the deformity during the follow up, we will plan for operative management in the form of flexor digitorum superficialis (FDS) tenotomy, or FDS transfer to radial lateral band.

**Figure 1:**
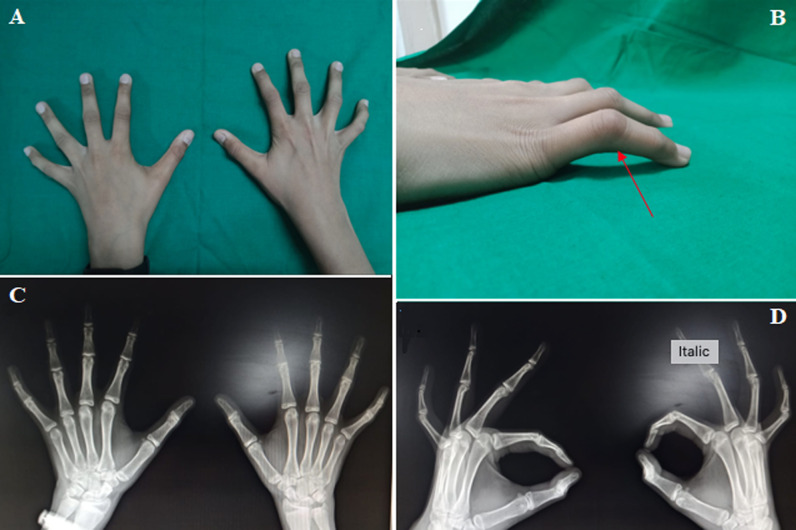
(A) clinical photograph of the both hands showing flexion contracture in the bilateral ring finger at the level of PIP joint; (B) lateral view of the right hand showing the flexion contracture at the level of PIP joint (less than 30 degree), marked with red colored arrow; (C) anteroposterior view of the both hand showing normal bony alignment; (D) oblique view of both hand showing normal bony alignment

